# Supplementary material for: Protein design and variant prediction using autoregressive generative models
Source: Nat Commun. 2021 Apr 23;12:2403. doi: 10.1038/s41467-021-22732-w (PMC8065141; doi:10.1038/s41467-021-22732-w)
Supplement: Supplementary file 1 — Supplementary Information [file 41467_2021_22732_MOESM1_ESM.pdf]

Protein Design and Variant Prediction Using Autoregressive Generative Models
Supplementary Information

This file includes:

Supplementary Figures 1 to 12
Supplementary Tables 1 to 6

Supplementary Figures

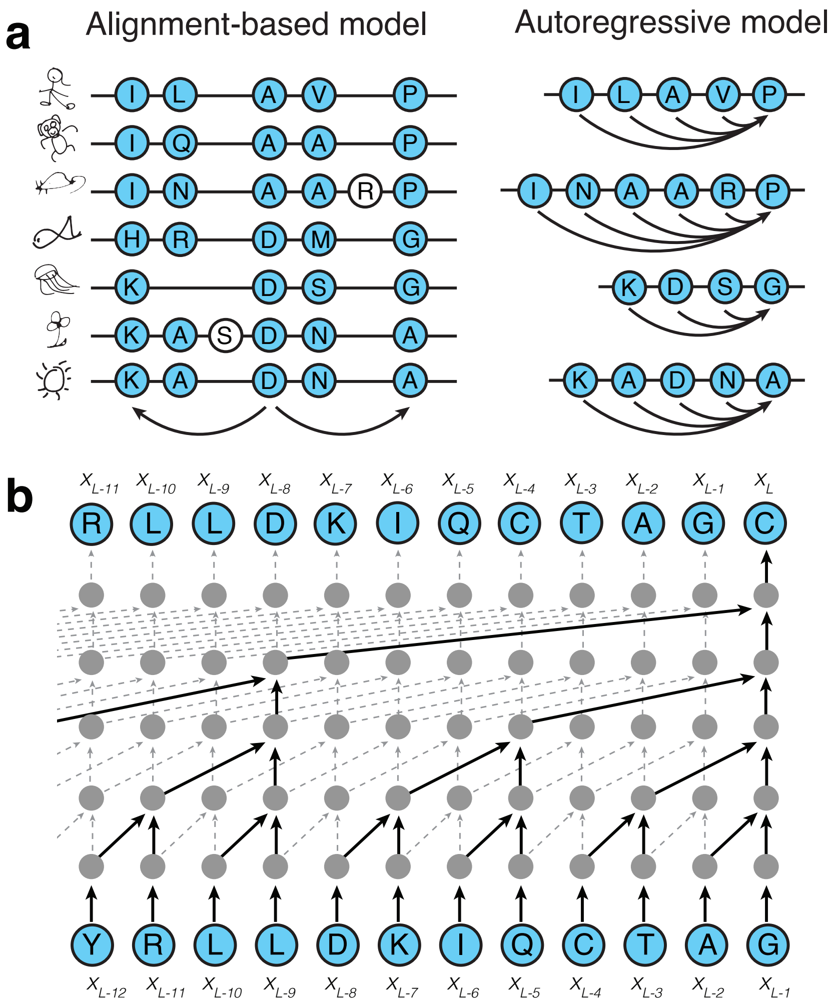

Supplementary Fig. 1. Autoregressive models of biological sequences. **a** Instead of finding correlations between columns in a multiple sequence alignment (left), the autoregressive model predicts a residue given all the preceding positions (right). **b** Causal dilated convolutions are used to model the autoregressive likelihood.

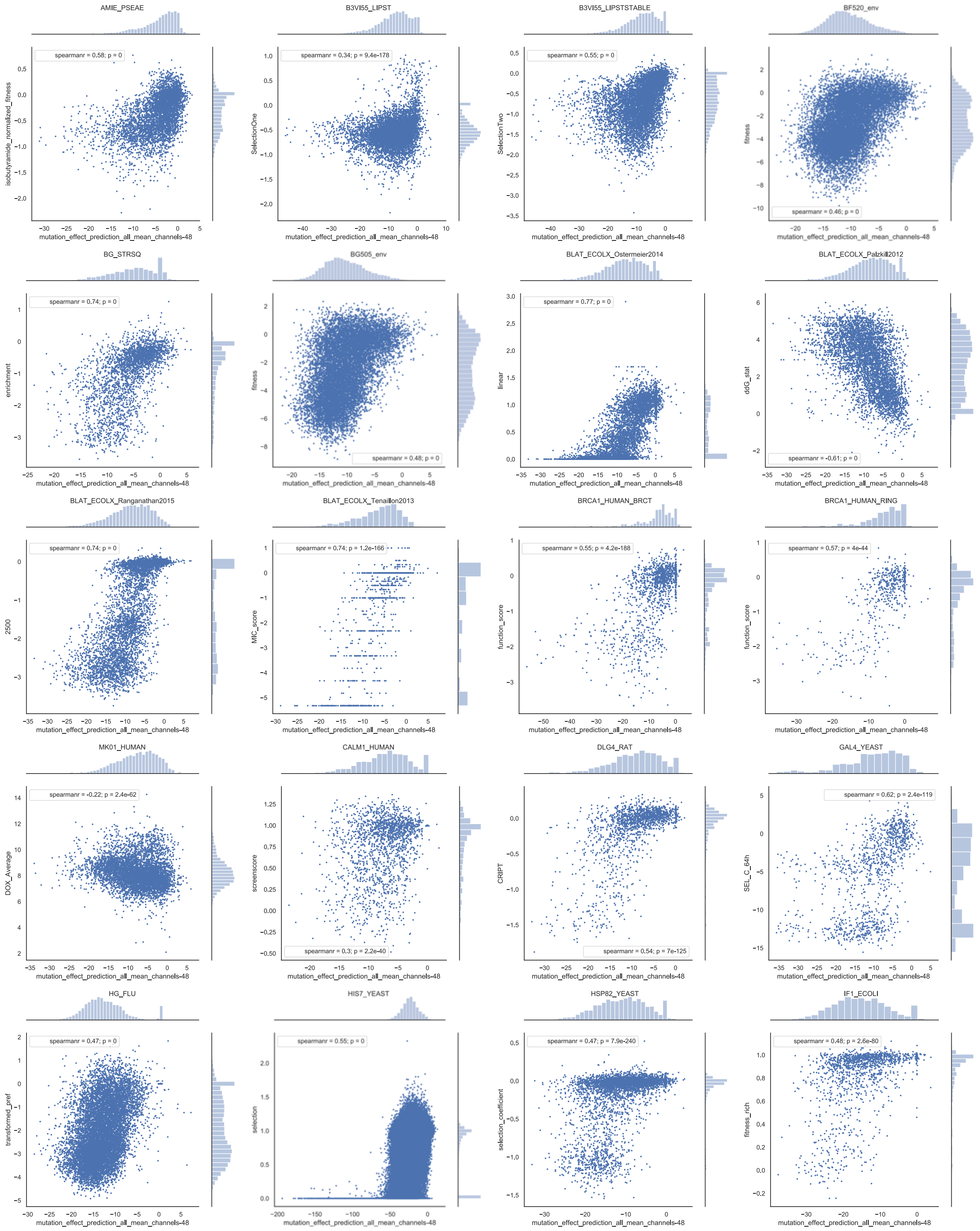
AMIE_PSEAE
spearmanr = 0.58; p = 0
isobutyramide_normalized_fitness
B3VI55_LIPST
spearmanr = 0.34; p = 9.4e-178
SelectionOne
B3VI55_LIPSTSTABLE
spearmanr = 0.55; p = 0
SelectionTwo
BF520_env
spearmanr = 0.46; p = 0
fitness
BG_STRSQ
spearmanr = 0.74; p = 0
enrichment
BG505_env
spearmanr = 0.48; p = 0
fitness
BLAT_ECOLX_Ostermeier2014
spearmanr = 0.77; p = 0
linear
BLAT_ECOLX_Palzkill2012
spearmanr = -0.61; p = 0
ddG_stat
BLAT_ECOLX_Ranganathan2015
spearmanr = 0.74; p = 0
2500
BLAT_ECOLX_Tenaillon2013
spearmanr = 0.74; p = 1.2e-166
MIC_score
BRCA1_HUMAN_BRCT
spearmanr = 0.55; p = 4.2e-188
function_score
BRCA1_HUMAN_RING
spearmanr = 0.57; p = 4e-44
function_score
MK01_HUMAN
spearmanr = -0.22; p = 2.4e-62
DOX_Average
CALM1_HUMAN
spearmanr = 0.3; p = 2.2e-40
screenscore
DLG4_RAT
spearmanr = 0.54; p = 7e-125
CRIPT
GAL4_YEAST
spearmanr = 0.62; p = 2.4e-119
SEL_C_64h
HG_FLU
spearmanr = 0.47; p = 0
transformed_pref
HIS7_YEAST
spearmanr = 0.55; p = 0
selection
HSP82_YEAST
spearmanr = 0.47; p = 7.9e-240
selection_coefficient
IF1_ECOLI
spearmanr = 0.48; p = 2.6e-80
fitness_rich
mutation_effect_prediction_all_mean_channels-48

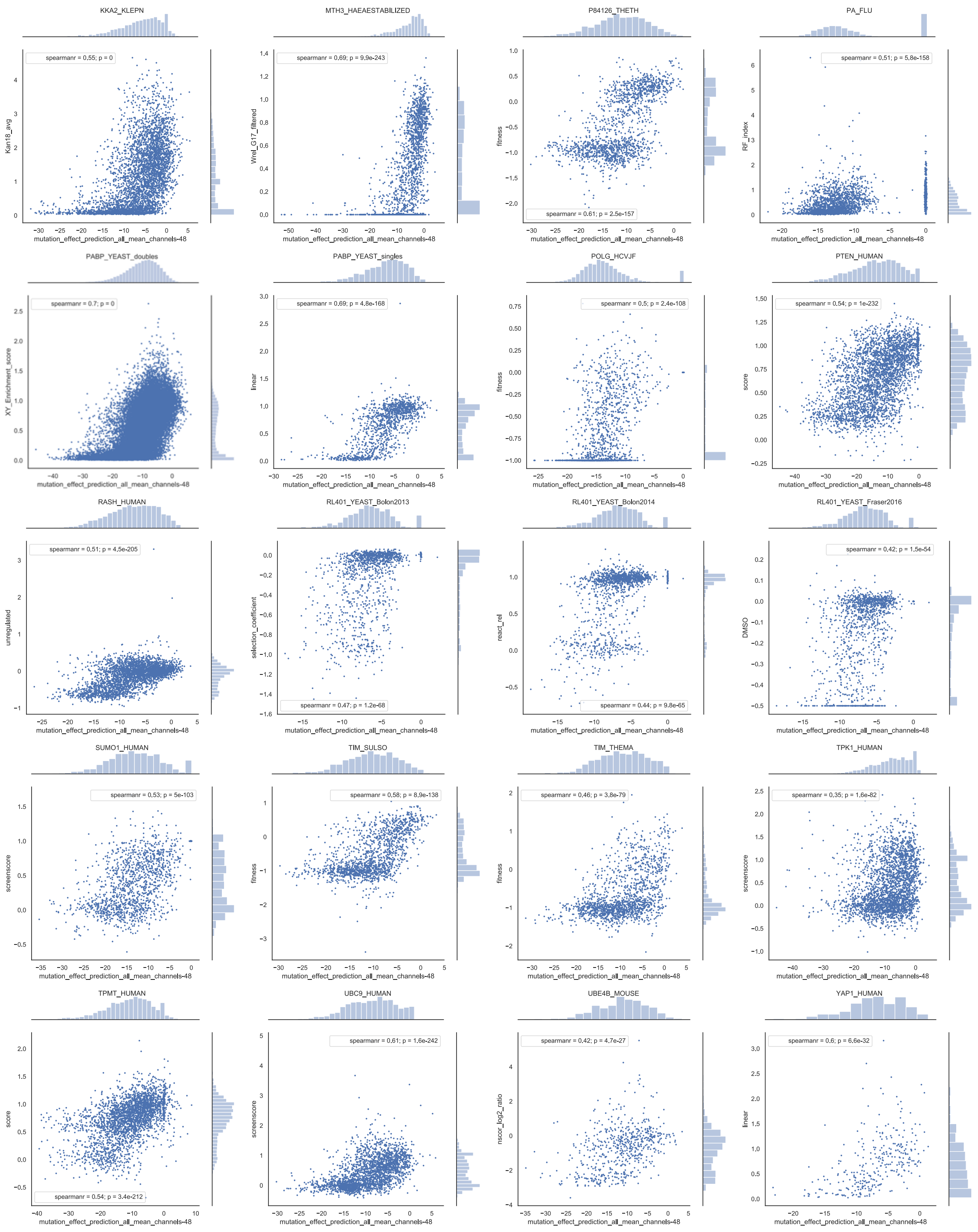

Supplementary Fig. 2. Individual scatterplots of the experimental results and the mutation effect prediction using the autoregressive model trained on each individual family of naturally occurring proteins. Experimental measures of fitness are on the y-axis and fitness predictions are on the x-axis. Spearman correlation values and corresponding two-tailed p-values are displayed per plot.

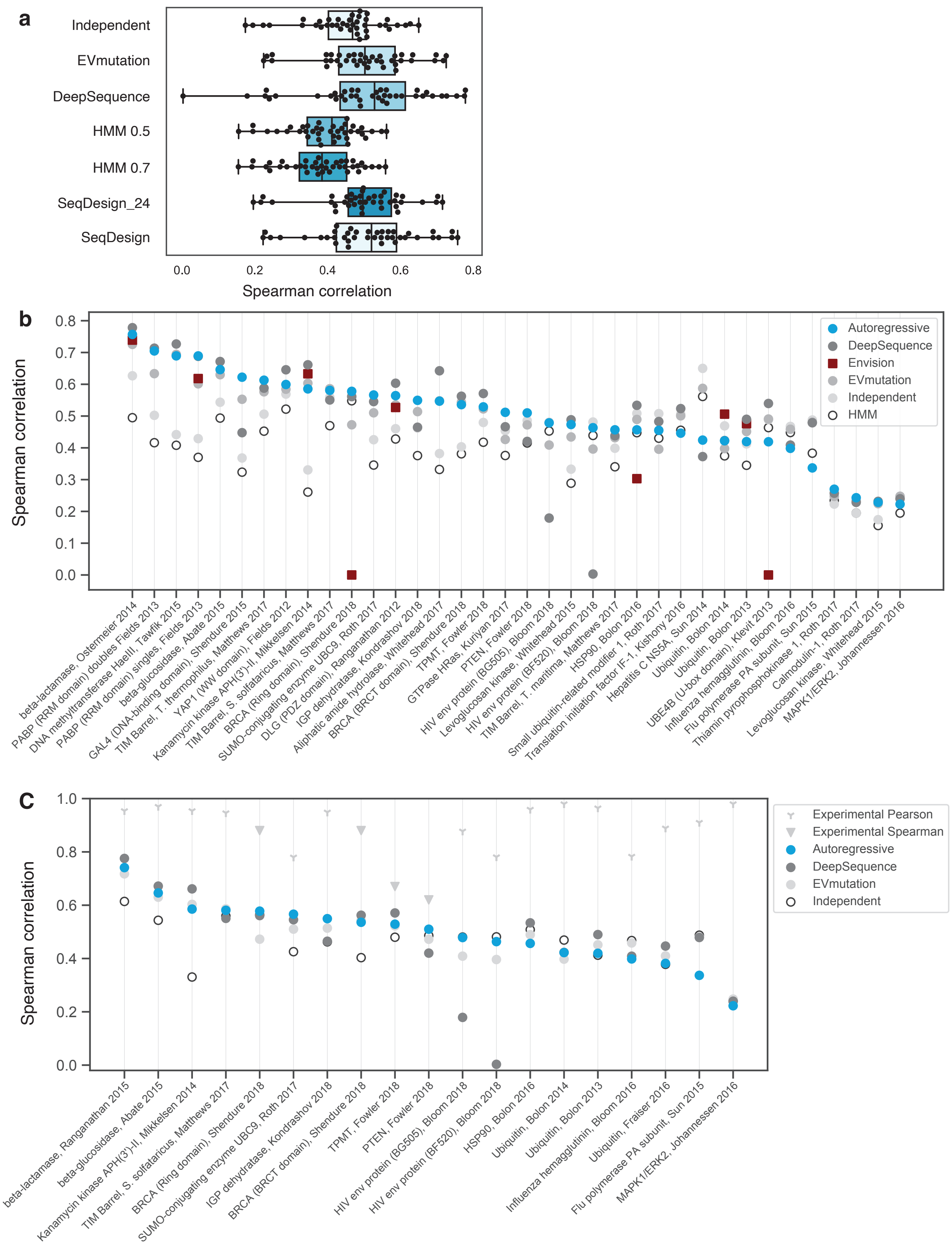
a
Independent
EVmutation
DeepSequence
HMM 0.5
HMM 0.7
SeqDesign_24
SeqDesign
0.0
0.2
0.4
0.6
0.8
Spearman correlation
b
Spearman correlation
0.8
0.7
0.6
0.5
0.4
0.3
0.2
0.1
0.0
Autoregressive
DeepSequence
Envision
EVmutation
Independent
HMM
beta-lactamase, Ostermeier 2014
PABP (RRM domain) doubles Fields 2013
DNA methyltransferase HaeIII, Tawfik 2015
PABP (RRM domain) singles, Fields 2013
beta-glucosidase, Abate 2015
GAL4 (DNA-binding domain), Shendure 2015
TIM Barrel, T. thermophilus, Matthews 2017
YAP1 (WW domain), Fields 2012
Kanamycin kinase APH(3')-II, Mikkelsen 2014
TIM Barrel, S. solfataricus, Matthews 2017
BRCA (Ring domain), Shendure 2018
SUMO-conjugating enzyme UBC9, Roth 2017
DLG (PDZ domain), Ranganathan 2012
IGP dehydratase, Kondrashov 2018
Aliphatic amide hydrolase, Whitehead 2017
BRCA (BRCT domain), Shendure 2018
TPMT, Fowler 2018
GTPase HRas, Kuriyan 2017
PTEN, Fowler 2018
HIV env protein (BG505), Bloom 2018
Levoglucosan kinase, Whitehead 2015
HIV env protein (BF520), Bloom 2018
TIM Barrel, T. maritima, Matthews 2017
HSP90, Bolon 2016
Small ubiquitin-related modifier 1, Roth 2017
Translation initiation factor IF-1, Kishony 2016
Hepatitis C NS5A, Sun 2014
Ubiquitin, Bolon 2014
Ubiquitin, Bolon 2013
UBE4B (U-box domain), Klevit 2013
Influenza hemagglutinin, Bloom 2016
Flu polymerase PA subunit, Sun 2015
Thiamin pyrophosphokinase 1, Roth 2017
Calmodulin-1, Roth 2017
Levoglucosan kinase, Whitehead 2015
MAPK1/ERK2, Johannessen 2016
C
Spearman correlation
1.0
0.8
0.6
0.4
0.2
0.0
Experimental Pearson
Experimental Spearman
Autoregressive
DeepSequence
EVmutation
Independent
beta-lactamase, Ranganathan 2015
beta-glucosidase, Abate 2015
Kanamycin kinase APH(3')-II, Mikkelsen 2014
TIM Barrel, S. solfataricus, Matthews 2017
BRCA (Ring domain), Shendure 2018
SUMO-conjugating enzyme UBC9, Roth 2017
IGP dehydratase, Kondrashov 2018
BRCA (BRCT domain), Shendure 2018
TPMT, Fowler 2018
PTEN, Fowler 2018
HIV env protein (BG505), Bloom 2018
HIV env protein (BF520), Bloom 2018
HSP90, Bolon 2016
Ubiquitin, Bolon 2014
Ubiquitin, Bolon 2013
Influenza hemagglutinin, Bloom 2016
Ubiquitin, Fraiser 2016
Flu polymerase PA subunit, Sun 2015
MAPK1/ERK2, Johannessen 2016

Supplementary Fig. 3. Comparison of model performance across datasets. **a** The Spearman correlation distributions of predictions for each model compared to 40 experimental datasets of deep mutational scans across 33 proteins, totaling 690,257. Two fragment lengths were used to build the HMM models (0.5 and 0.7), and only 0.5 is displayed in Figure 2. Two hidden sizes (24 and 48) were tested for the autoregressive model; 48 was chosen for further study. The box-plot elements are as follows: center line, median; box limits, upper and lower quartiles; whiskers, range of values. Mean and quartile values are displayed for each model in the box-and-whisker plot, whiskers span the entire range. **b** A comparison of model prediction Spearman correlations, including Envision LOPO prediction correlations. BRCA1 RING and UBE4B were validated but not tested by Envision due to poor validation performance. **c** A comparison of model predictions' Spearman correlation with every dataset for which experimental Spearman or Pearson correlations are available for biological or technical replicates. Pearson correlations have been included where a Spearman correlation is unavailable.

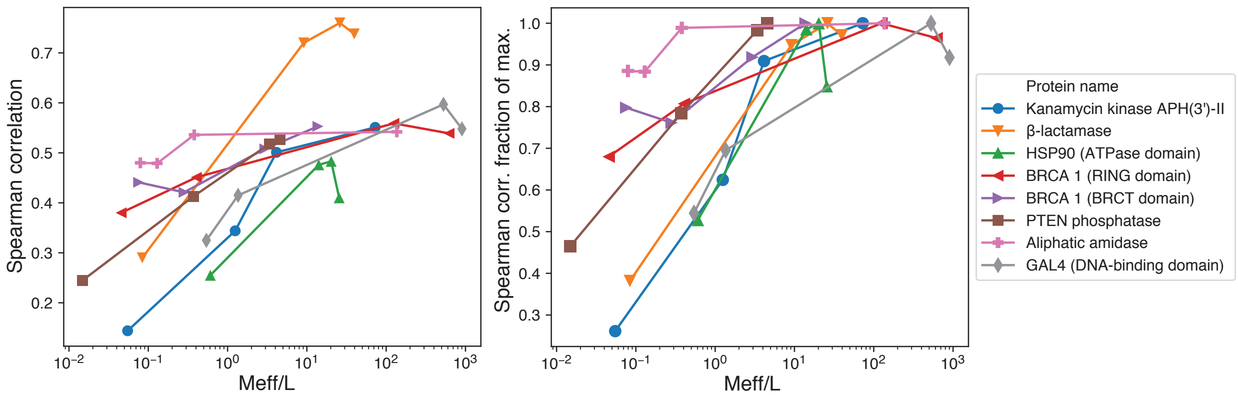

Supplementary Fig. 4. Spearman correlations for models trained on alignments of 8 protein families at 4 depths. Spearman correlations shown as-is (left) or normalized to the highest observed correlation for each family (right). Meff/L is the length-normalized number of nonredundant sequences after weighting sequences at 80% identity. Prediction accuracy for aliphatic amidase is nearly identical between a 151,555 sequence set (Meff=36,020; Meff/L=136; ρ=0.542) and a 3,982 sequence set (Meff=123; Meff/L=0.38; ρ=0.536).

Autoregressive:

Zabetakis, et al (2013)

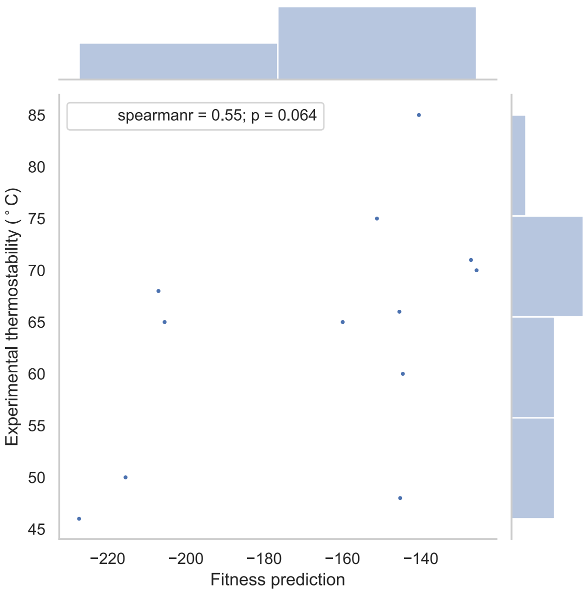

Kunz, et al (2017)

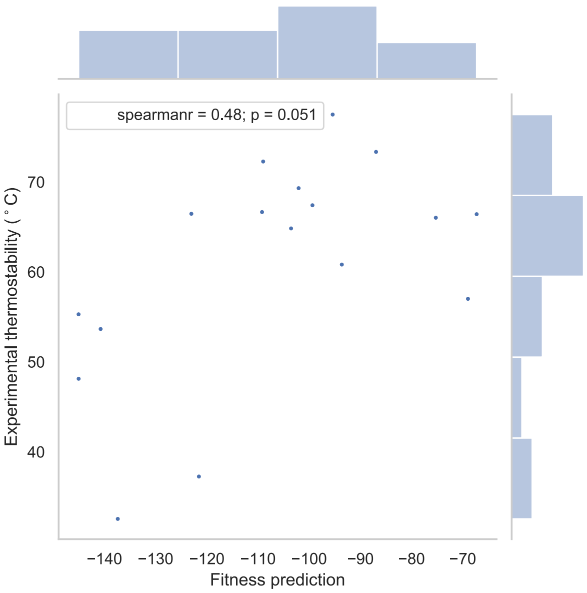

Shriver-Lake, et al (2017)

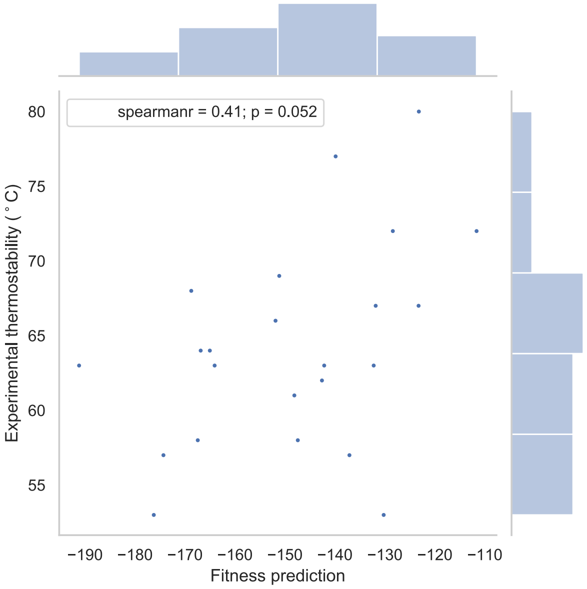

Turner, et al (2015)

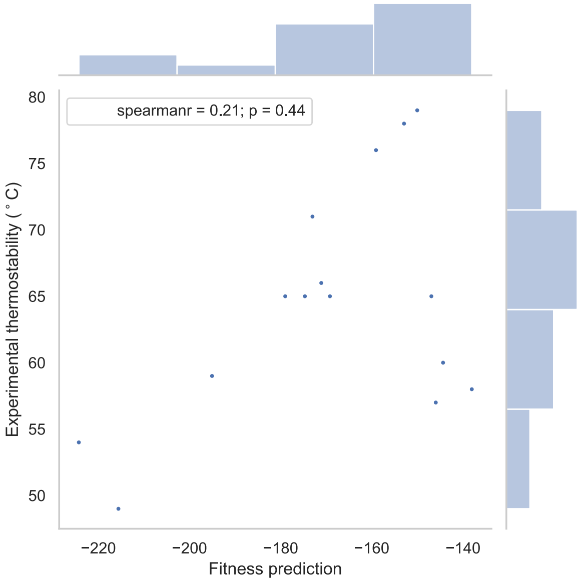

HMM:

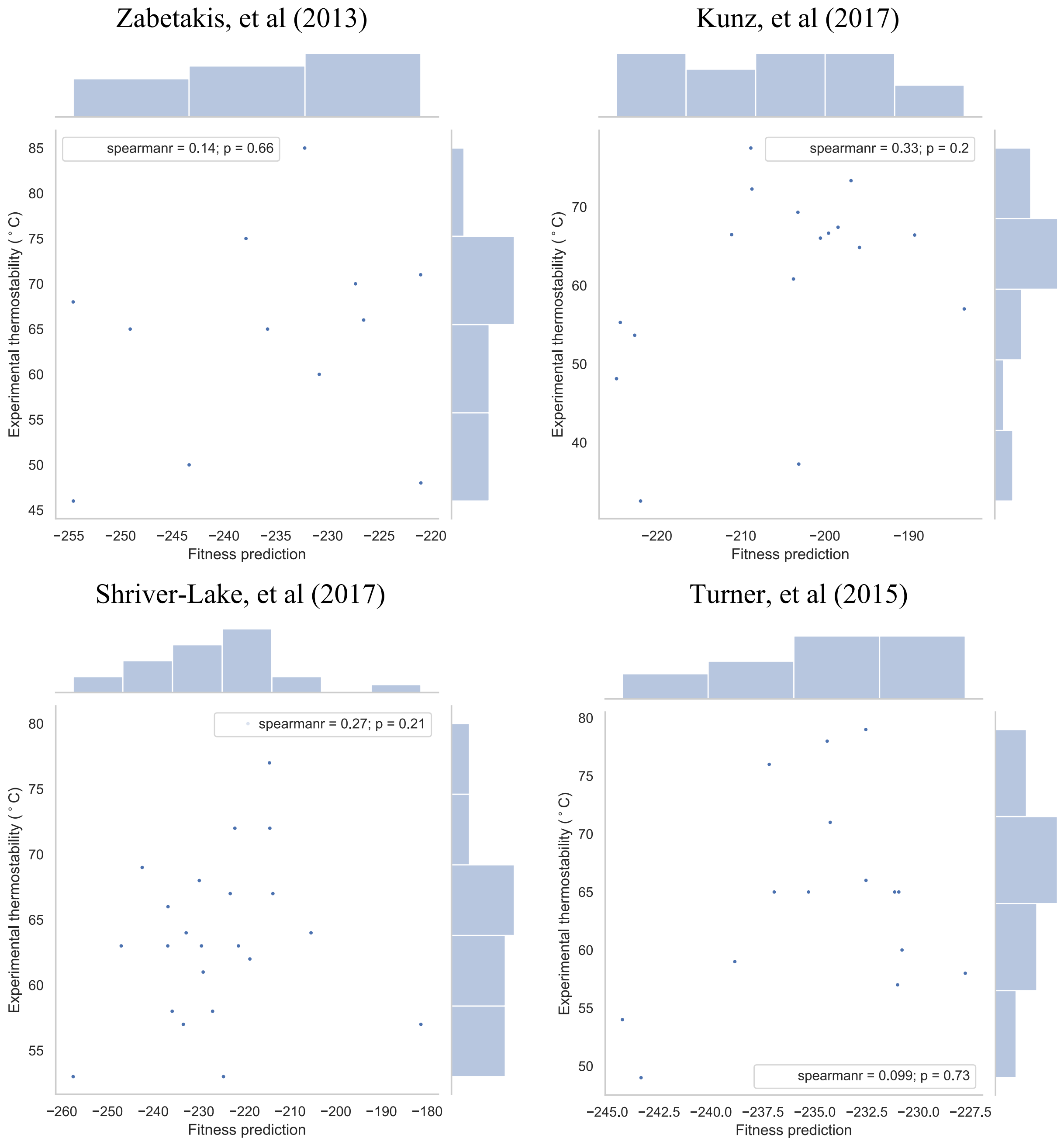

Supplementary Fig. 5. Fitness prediction of the autoregressive and HMM models vs. experimental thermostability measurements from nanobody thermostability datasets for which there are at least ten data points. Thermostability measurements are in degrees Celsius and the fitness predictions are reported as log probability scores of each nanobody sequence. Spearman correlation values and corresponding two-tailed p-values are displayed per plot.

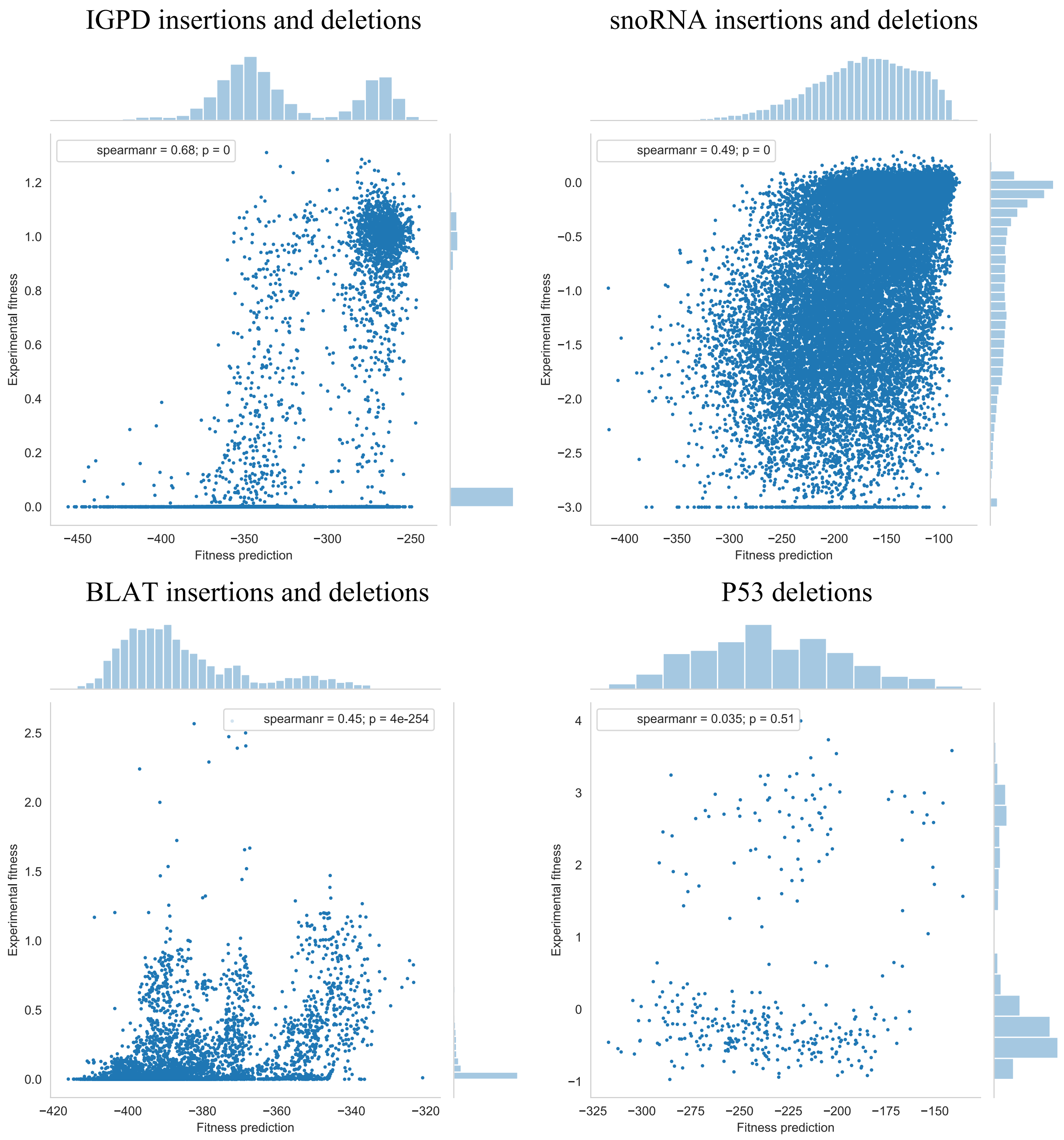

Supplementary Fig. 6. Indel mutation scan measurement comparisons for three proteins and one RNA: IGP dehydratase, snoRNA, β-lactamase and P53. Spearman correlation values and corresponding two-tailed p-values are displayed per plot.

APOE

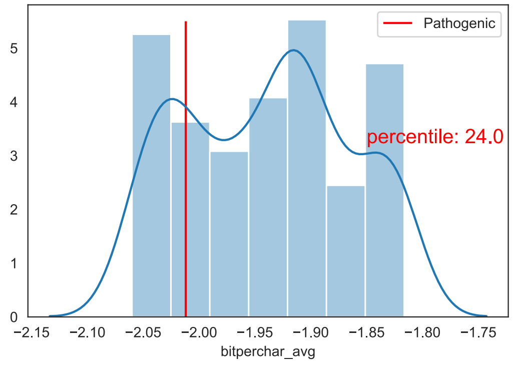

P53

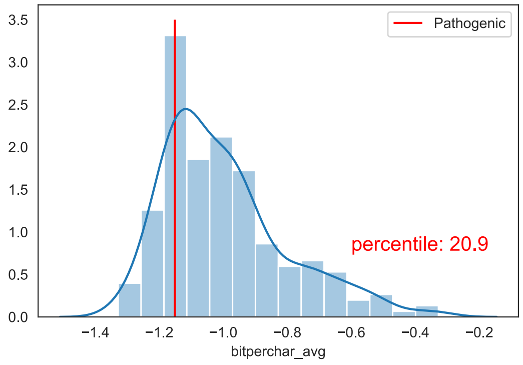

BRCA1 (BRCT region)

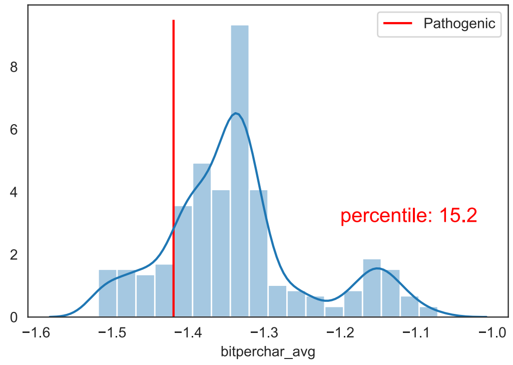

BRCA1 (RING region)

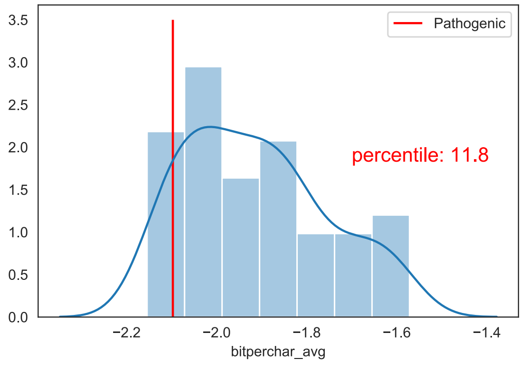

Supplementary Fig. 7. Pathogenic single amino acid deletions (as annotated by Clinvar) are predicted to be on the more deleterious spectrum of all possible single amino acid deletion effect predictions in a gene indicated in Alzheimer's (APOE), and two genes indicated in cancer (P53, BRCA1). Other single amino acid deletions that are predicted to be highly deleterious by the autoregressive model may be interesting to test for pathogenicity.

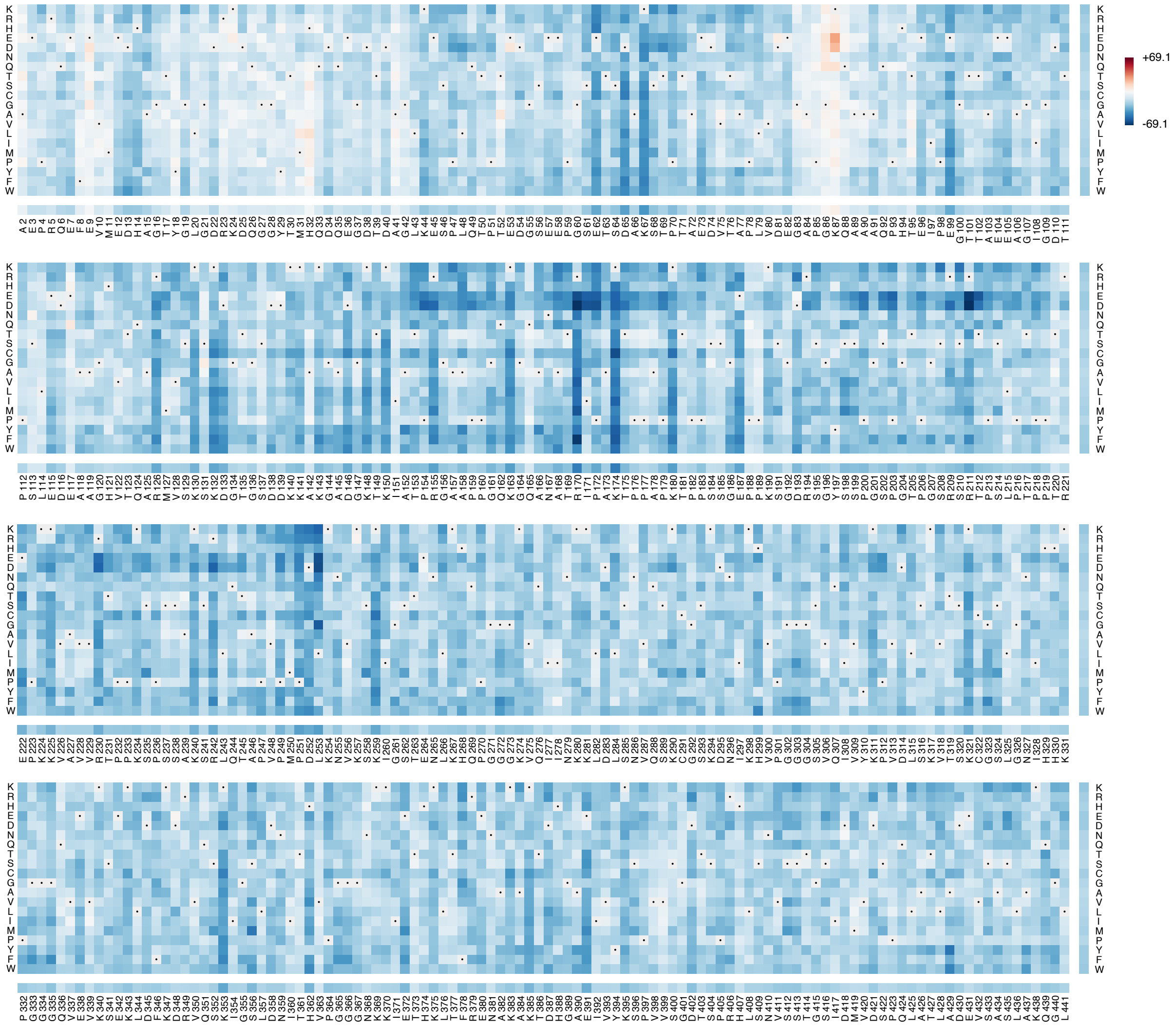
+69.1
-69.1

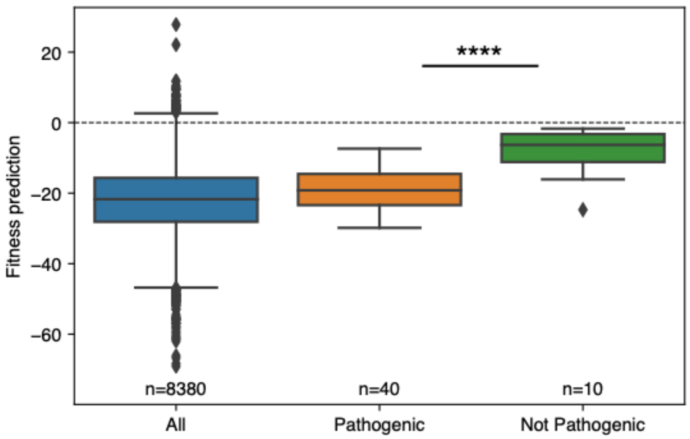
****
20
0
−20
−40
−60
Fitness prediction
n=8380
n=40
n=10
All
Pathogenic
Not Pathogenic

Supplementary Fig. 8. (top) *In silico* mutation scan of all single mutants for the human Tau protein, isoform Tau-4 (P10636-8). (bottom) Distribution of fitness predictions relative to wild-type for all mutations and for known variants annotated as pathogenic or not pathogenic in the Alzforum repository (https://www.alzforum.org/mutations/mapt). The box-plot elements are as follows: center line, median; box limits, upper and lower quartiles; whiskers, 1.5x interquartile range; points, outliers. Fitness predictions distinguish pathogenic and not pathogenic groups (two-tailed independent t=-4.5, p=3.8×10^{-5} (****, $p < 0.0001$); AUC=0.86).

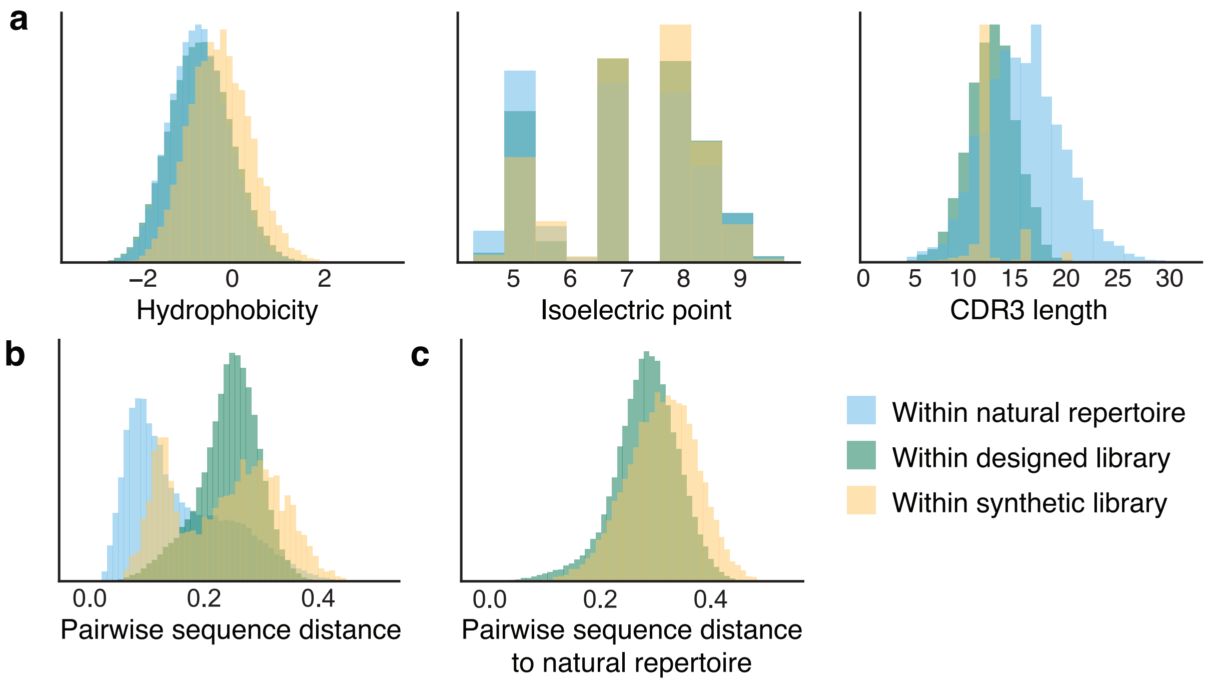

Supplementary Fig. 9. Compared to a synthetic library generated by codon randomization (McMahon et al., 2018), the designed library has more similar properties to the natural repertoire, while maintaining nearly the diversity of the synthetic library. **a** Synthetic CDR3s have slightly different distributions of hydrophobicity and isoelectric point from the natural repertoire, and the synthetic library contains CDR3s of 3 lengths rather than a broad length distribution. **b**, **c** The designed library contains nearly as much diversity as the synthetic library as measured by **b**, distances to the nearest neighbors within each library, and **c**, distances to the nearest neighbors in the natural llama single-domain antibody repertoire.

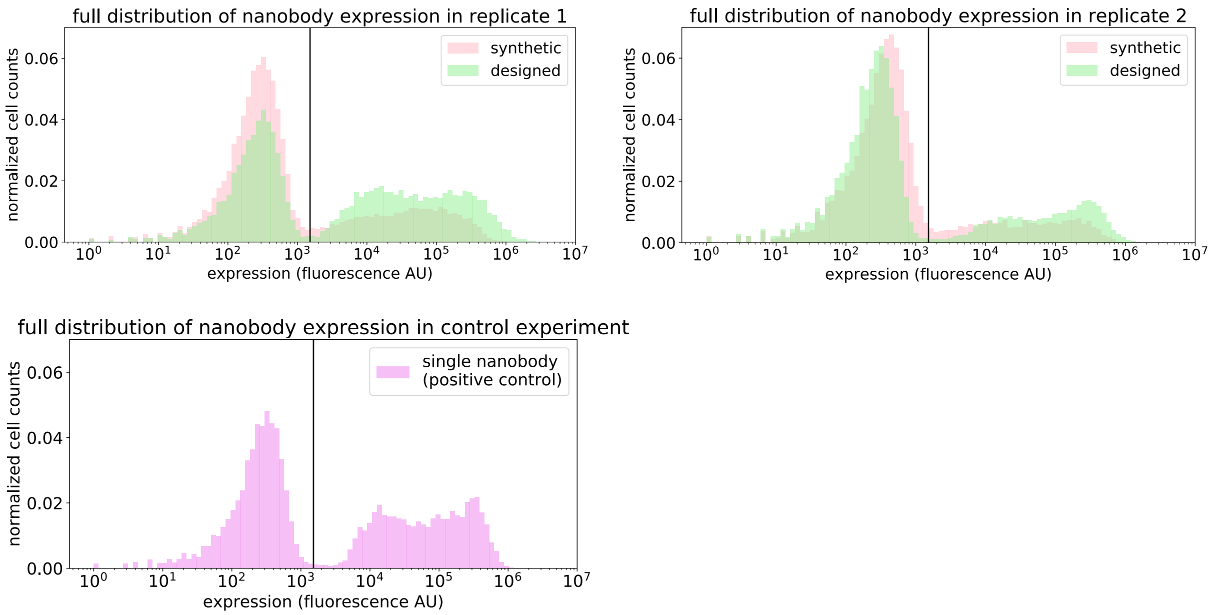

Supplementary Fig. 10. Full distributions of nanobody expression in the original synthetic library and our designed library. The designed library has a larger fraction of cells expressing nanobodies compared to the synthetic library (large difference in replicate 1 and small in replicate 2) and is closer to resembling the positive control. The vertical lines are the local minima between the non-expressing cells (the mode to the left of the line) and the expressing cells (to the right of the line). The distribution of the expressing cells are displayed in the main text in Fig. 4a.

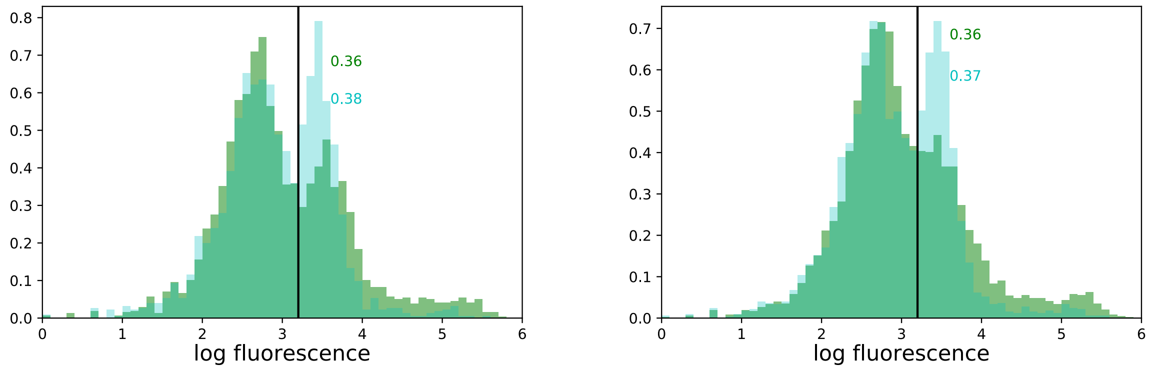

Supplementary Fig. 11. Experimental measurements of polyreactivity in the designed library (green) as compared to the original synthetic library (blue) shows similar, if not slightly lower proportions of poly-reactive nanobodies in the designed library when sorted for binding to a non-specific insect cell membrane reagent. The log experimental fluorescent measurements are shown on the x-axis, in two replicates (left and right).

Design nanobody library

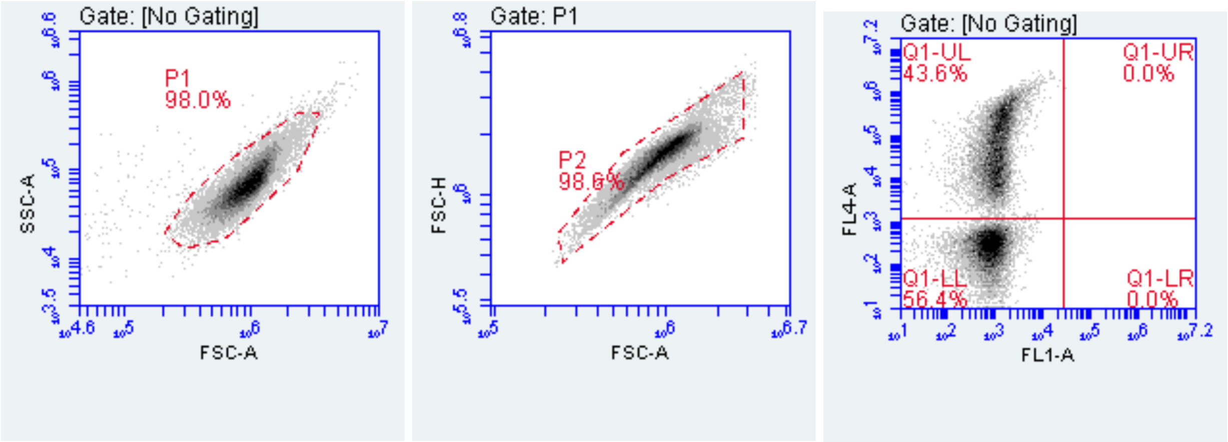

Synthetic nanobody library (McMahon, et al, 2018)

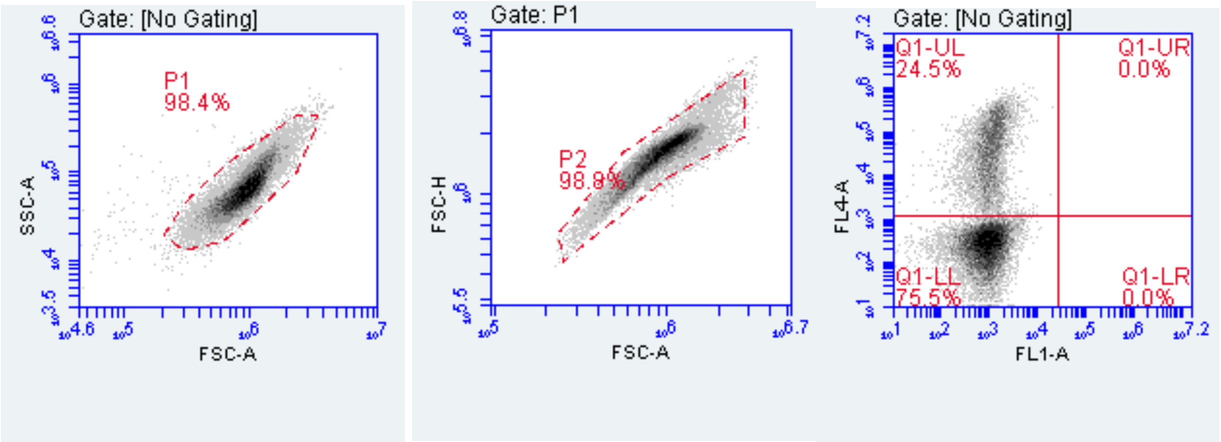

Supplementary Fig. 12. Example of flow cytometer yeast gating. Standard gating was used. Yeast form a single population in an FSC/SSC plot and were gated accordingly. Cells were further gated on an FSC-A/ FSC-H plot to exclude doublets. FL1-A uses AlexaFluor 647 and measures expression of the nanobodies per cell (Fig. 4a,b, Supplementary Fig. 10). FL4-A uses AlexaFluor 488 and is used to measure binding to antigen (HSA: Fig. 4c,d; PSR (poly-reactivity reagent): Supplementary Fig. 11).

Supplementary Tables

Protein name	Uniprot ID (Nov 2015)	Measurement	# Mutations (mutant type)	Coverage	Number of replicates	Replicate correlations	Correlation notes	Reference
β-glucosidase	(Sequence from dataset)	Enzyme function	3000 (single)	1-501	2	0.97	Pearson R, from paper	Romero et al., PNAS, 2015
β-lactamase	BLAT_ECOLX	Growth	3000 (single)	24-286	1	n/a	they report an estimated error per measureme nt	Firnberg et al., Mol Biol Evol, 2014
β-lactamase	BLAT_ECOLX	Growth	4997 (single)	26-290	2	0.91	Pearson R^2, from paper	Stiffler et al., Cell, 2015
β-lactamase	BLAT_ECOLX	MIC	990 (single)	24-286	1	n/a	Two experiment al conditions reported, 1 replicate each	Jacquier et al., PNAS 2013
β-lactamase	BLAT_ECOLX	Growth	4998 (single)	24-286	1	n/a	One replicate provided	Deng et al., JMB, 2012
PSD95 (PDZ domain)	DLG4_RAT	Peptide binding	1578 (single)	311-393	1	n/a	One replicate provided	McLaughlin et al., Nature, 2012
GAL4 (DNA-binding domain)	GAL4_YEAST	Growth	1196 (single)	2-65	1	n/a	One replicate provided	Kitzman et al., Nat Methods, 2015
Influenza hemagglutinin	(Sequence from paper)	Viral replication	10717 (single)	2-565	3	0.66, 0.59, 0.59	Pearson R^2, from paper	Doud & Bloom, Viruses, 2016
HSP90 (ATPase domain)	HSP82_YEAST	Growth	4324 (single)	2-231	2	0.92	Pearson R^2, from paper, but only for 20 amino acid positions	Mishra et al., Cell Reports, 2016
Kanamycin kinase APH(3')-II	KKA2_KLEPN	Growth	4582-4996 (single)	1-264	2	0.91	Pearson R, from paper	Melnikov et al., NAR, 2014
DNA methylase HaeIII	(stabilized sequence based on MTH3_HAEAE)	Growth	1957 (single, full), 1778 (single, filtered)	2-330	1	n/a	One replicate provided	Rockah-Shmuel et al., PLOS Comp Bio, 2015
Influenza polymerase PA subunit	(Sequence from paper)	Viral replication	1822 (single)	8-716	2	0.96	Pearson R, from paper	Wu et al., PLOS Genetics, 2015
Poly(A)-binding protein (RRM domain)	PABP_YEAST	Growth	1188 (single), 36522 (double)	126-200	1	n/a	One replicate provided	Melamed et al., RNA, 2013
Hepatitis C NS5A	POLG_HCVJF	Viral replication	1632 (single)	1994-2097	1	n/a	One replicate provided	Qi et al., PLOS Pathogens, 2014
Ubiquitin	RL401_YEAST	Growth	1196 (single)	2-76	2	0.93	Pearson R^2, from paper	Roscoe et al, JMB, 2013
Ubiquitin	RL401_YEAST	E1 reactivity	1366 (single)	2-76	2	0.96	Pearson R^2, from paper	Roscoe et al, JMB, 2014
UBE4B (U-box domain)	UBE4B_MOUSE	Ligase activity	900 (single)	1072-1173	1	n/a	One replicate provided	Starita et al., PNAS, 2013

YAP1 (WW domain 1)	YAP1_HUMAN	Peptide binding	363 (single)	170-203	1	n/a	One replicate provided	Araya et al., PNAS, 2012
Aliphatic amidase	AMIE_PSEAE	Enzyme function	4507-4554 (single)	1-341	1	0.889	Pearson R, from paper	Wrenbeck et al., Nat Commun, 2017
TIM Barrell (T. thermophilus)	P84126_THETH	Growth	1520 (single)	44-238	1	n/a	One replicate provided	Chan et al., Nat Commun, 2017
TIM Barrell (S. solfataricus)	TRPC_SULSO	Growth	1520 (single)	44-235	2	0.946	Pearson R, from paper, but only for 20 amino acid positions	Chan et al., Nat Commun, 2017
TIM Barrell (T. maritima)	TRPC_THEMA	Growth	1520 (single)	40-230	1	n/a	One replicate provided	Chan et al., Nat Commun, 2017
Translation initiation factor IF-1	IF1_ECOLI	Growth	1274 (single)	1-72	1	n/a	One replicate provided	Kelsic et al., Cell Systems, 2016
Mitogen-activated protein kinase 1	MK01_HUMAN	Growth	5463 (single)	2-360	4-6	0.98	Pearson R, calculated from raw data	Brenan et al., Cell Rep, 2016
Hras	RASH_HUMAN	Enzyme function	3040 (single)	2-166	1	n/a	One replicate provided	Bandaru et al., Elife, 2017
Ubiquitin	RL401_YEAST	Growth	1142-1201 (single)	2-76	2	0.79	Pearson R^2, from paper	Mavor et al., Elife, 2016
BRCA 1 (RING domain)	BRCA1_HUMAN	Growth	492 (single)	1631-1855	2	0.88	Spearman R, from paper	Findlay et al., Nature 2018
BRCA 1 (BRCT domain)	BRCA1_HUMAN	Growth	1185 (single)	1-101	2	0.88	Spearman R, from paper	Findlay et al., Nature 2018
Thiopurine S-methyltransferase	TPMT_HUMAN	Protein stability	2659 (single)	1-245	8	0.67	Spearman R, from paper	Matreyek et al., Nat Gen, 2019
Phosphatidylinositol 3,4,5-trisphosphate 3-phosphatase and dual-specificity protein phosphatase PTEN	PTEN_HUMAN	Protein stability	3014 (single)	2-403	8	0.62	Spearman R, from paper	Matreyek et al., Nat Gen, 2019
Levoglucosan kinase (stabilized)	(Stabilized sequence based on B3VI55_LIPST)	Growth	6327 (single)	1-439	2	n/a	Plot provided, but no correlation metric	Klesmith et al., ACS Synth Bio, 2015
Levoglucosan kinase	B3VI55_LIPST	Growth	6541 (single)	1-439	2	n/a	Plot provided, but no correlation metric	Klesmith et al., ACS Synth Bio, 2015
HIV env protein (BF520)	(Sequence from paper)	Viral replication	12236 (single)	30-691	3	0.59,0.60,0.64	Pearson R, from paper	Haddox et al. Elife, 2018
HIV env protein (BG505)	(Sequence from paper)	Viral replication	12217 (single)	30-699	3	0.76,0.77,0.78	Pearson R, from paper	Haddox et al. Elife, 2018
Imidazoleglycerol-phosphate dehydratase (His3)	HIS7_YEAST	Growth	496137 (1-28 mutations)	1-220	2	0.9	Pearson R^2, from paper	Pokusaeva et al., PLOS Genetics, 2019 (bioRxiv 2017)
Calmodulin-1	CALM1_HUMAN	Yeast growth	1730 (single)	2-149	1	n/a	One replicate provided	Weile et al., MSB, 2017

Thiamin pyrophosphokinase 1	TPK1_HUMAN	Yeast growth	2608 (single)	2-243	1	n/a	One replicate provided	Weile et al., MSB, 2017
Small ubiquitin-related modifier 1	SUMO1_HUMAN	Yeast growth	1329 (single)	2-101	1	n/a	One replicate provided	Weile et al., MSB, 2017
SUMO-conjugating enzyme UBC9	UBC9_HUMAN	Yeast growth	2281 (single)	2-158	2	0.78	Pearson R, from paper	Weile et al., MSB, 2017

Supplementary Table 1. Deep mutational scans included in the paper and information regarding the experimental data, including sequence information, number of mutations, and experimental replicate measurements.

Protein name	Uniprot ID	Original # sequences	Original Meff	Bitscore	# sequences	Meff	Sequence coverage	Meff/L	Original spearman	Spearman correlation
Kanamycin kinase APH(3')-II	KKA2_KLEPN	29705	9380	0.3	56823	17439	240	72.66	0.554	0.551
				0.5	4314	1034	250	4.14		0.501
				1	1636	318	255	1.25		0.344
				1.5	119	14	255	0.05		0.144
β-lactamase	BLAT_ECOLX	14691	3667	0.3	40391	10013	253	39.58	0.741	0.738
				0.5	27648	6691	257	26.04		0.760
				1	14614	2346	257	9.13		0.720
				1.5	1539	22	262	0.08		0.290
HSP90 (ATPase domain)	HSP82_YEAST	23260	2586	0.3	45433	5672	222	25.55	0.473	0.410
				0.5	43521	4517	223	20.26		0.483
				1	32922	3176	225	14.12		0.476
				1.5	4719	137	226	0.61		0.255
BRCA 1 (RING domain)	BRCA1_HUMAN	39129	6304	0.3	251665	37215	60	620.25	0.571	0.539
				0.5	78851	9508	76	125.11		0.559
				1	1125	44	109	0.40		0.451
				1.5	845	5	110	0.05		0.380
BRCA 1 (BRCT domain)	BRCA1_HUMAN	8331	1883	0.3	13773	2829	205	13.80	0.545	0.553
				0.5	3272	635	215	2.95		0.508
				1	1099	63	223	0.28		0.421
				1.5	808	17	225	0.08		0.441
PTEN phosphatase	PTEN_HUMAN	8487	994	0.3	15397	1475	323	4.57	0.536	0.527
				0.5	13168	1100	325	3.38		0.518
				1	1314	146	392	0.37		0.413
				1.5	709	6	403	0.01		0.245
Aliphatic amidase	AMIE_PSEAE	76187	19554	0.3	151555	36020	264	136.44	0.578	0.542
				0.5	3982	123	324	0.38		0.536
				1	2145	44	340	0.13		0.479
				1.5	2087	27	340	0.08		0.480
GAL4 (DNA-binding domain)	GAL4_YEAST	22980	7026	0.3	156727	42406	47	902.26	0.622	0.548
				0.5	98386	25868	49	527.92		0.597
				1	354	91	67	1.36		0.415
				1.5	101	40	74	0.54		0.325

Supplementary Table 2. Spearman correlations of predictions with experiments using training sequence sets derived from alignments at four different bitscores. Meff is the number of nonredundant sequences after weighting sequences at 80% identity. Sequence coverage is the length of the focus sequence covered by the alignment, including non-focus columns that would have been excluded by alignment-based models.

Reference	Dataset description	Number of datapoints
Zabetakis, et al., PLoS One, 2013	Different regions stitched together	12
Shriver-Lake, et al., Toxicon, 2017	Various point mutations introduced in different frameworks	23
Turner, et al., Biotechnol Rep, 2015	Variety of mutation types (15 without disulfide linkage)	19
Kunz, et al., Biochim Biophys, 2017	Potentially stabilizing variants designed based on sequence analysis	17

Supplementary Table 3. Description of thermostability datasets for llama nanobody sequences used to validate the model's predictive capacity for nanobodies.

Protein name	Uniprot ID	# Mutations	Replicate correlations	Correlation notes	Reference
PTEN	PTEN_HUMAN	340 (deletions)	0.58		Mighell et al., Am. J. Hum. Genet., 2018
HIS3	HIS7_YEAST	5711 (deletions), 391 (insertions)	n/a	197 isolated strains show r = 0.82 for fitness as measured by competition vs growth rate	Pokusaeva et al., PLOS Genetics, 2019
snoRNA	CL00100 (RFAM)	3896 (insertions), 22772 (deletions), 33144 (missense)	0.87	many different measurements with different correlations, but the two with the same conditions: small, 30, glu has a correlation of 0.87	Puchta et al., Science, 2016
P53	P53_HUMAN	357 (deletions), 5858 (missense)	n/a	n/a	Kotler et al., Mol Cell, 2018
BLAT	BLAT_ECOLX	262 (deletions), 4422 (insertions)	n/a	n/a	Gonzalez et al., J Mol Biol, 2019

Supplementary Table 4. Indel mutation scans that were used for validating the model's predictive power for sequences of different lengths. Experimental scans are included in the paper and their summary statistics.

Step	Number of sequences (millions)
Generate sequences	33.0
Filter for reference final beta strand	23.6
Remove duplicate sequences	21.7
Remove duplicates of training set	6.25
Remove glycosylation motifs	6.11
Remove asparagine deamination motifs	6.04
Remove sulfur-containing amino acids	3.69

Supplementary Table 5. Number of unique nanobody sequences remaining after each step of filtering of the designed nanobody library.

Oligonucleotide name	Sequence
FragmentGENE_NbCM	GCTGCCCAGCCGGCGATGGCCCAGGTCCAACTTCAAGAATCAGGCGGGGGCCTGGTACAGGCAGGCGGTTCTCTTCGGCTGTCGTGTGCGGCAAGCGGATTTACATTCAGTAGCTACGCTATGGGCTGGTACCGTCAGGCACCGGGGAAAGAACGGGAATTTGTTGCTGCAATCTCTTGGAGCGGTGGGAGCACATATTATGCAGATTCCGTTAAAGGCAGATTCACGATCAGTCGCGATAACGCAAAAAATACAGTGTACTTACAAATGAACTCTTTGAAACCCGAAGACACCGCAGTCTATTACTGCGCGGCCGCTACTGGGGACAAGGCACCCAGGTGACTGTATCATCCCACCACCACCACCACCACTGA
NbCM_pydsF2.0	GGTGTTCAATTGGACAAGAGAGAAGCTGACGCAGAAGTCCAACTTGTCGAATCAGGCGGGGGCCTGGTACAG
NbCM_pydsR	CGTAATCTGGAACATCGTATGGGTAGGATCCGGATGATACAGTCACCTGGGT
NotI_removal_1F	CAACCCTCACTAAAGGGCGTTCGCCATGAGATTCCCATCTATCTTCA
Pyds_NbCM_cloning_ R	CACCTGGGTGCCTTGTCCCCAGTA

Supplementary Table 6. Oligonucleotides used for synthesis and construction for the nanobody library.
