# Supplementary material for: Protein design and variant prediction using autoregressive generative models
Source: Nat Commun. 2021 Apr 23;12:2403. doi: 10.1038/s41467-021-22732-w (PMC8065141; doi:10.1038/s41467-021-22732-w)
Supplement: Supplementary file 2 — Description of Additional Supplementary Files [file 41467_2021_22732_MOESM2_ESM.pdf]

Description of Additional Supplementary Files

File Name: Supplementary Data 1
Description: Nanobody thermostability prediction measurements with the autoregressive model trained on naïve llama nanobody sequences.

File Name: Supplementary Data 2
Description: PTEN phosphatase deletion predictions with the autoregressive model trained on the PTEN sequence family.

File Name: Supplementary Data 3
Description: IGP dehydratase insertion and deletion predictions with the autoregressive model trained on the IGP sequence family.

File Name: Supplementary Data 4
Description: snoRNA insertion and deletion predictions with the autoregressive model trained on the snoRNA clan from RFAM.

File Name: Supplementary Data 5
Description: Beta lactamase insertion and deletion predictions with the autoregressive model trained on the BLAT sequence family.

File Name: Supplementary Data 6
Description: P53 deletion predictions with the autoregressive model trained on the P53 sequence family.

File Name: Supplementary Data 7
Description: In silico mutation scan of the Tau protein using the autoregressive model to generate predictive scores for all single missense mutations, including the disordered repeat region of Tau, which is indicated in neurodegenerative disease.

File Name: Supplementary Data 8
Description: Predicted scores for Tau mutations categorized as pathogenic or not pathogenic in the Alzforum database. Mutations with uncertain significance were not included.
