# Supplementary material for: Protein design and variant prediction using autoregressive generative models
Source: Nat Commun. 2021 Apr 23;12:2403. doi: 10.1038/s41467-021-22732-w (PMC8065141; doi:10.1038/s41467-021-22732-w)
Supplement: Supplementary file 11 — Reporting Summary [file 41467_2021_22732_MOESM11_ESM.pdf]

nature research

Corresponding author(s): Debora Marks

Last updated by author(s): Mar 15, 2021

Reporting Summary

Nature Research wishes to improve the reproducibility of the work that we publish. This form provides structure for consistency and transparency in reporting. For further information on Nature Research policies, see Authors & Referees and the Editorial Policy Checklist.

Statistics

For all statistical analyses, confirm that the following items are present in the figure legend, table legend, main text, or Methods section.

n/a	Confirmed	
☐	☒	The exact sample size (n) for each experimental group/condition, given as a discrete number and unit of measurement
☒	☐	A statement on whether measurements were taken from distinct samples or whether the same sample was measured repeatedly
☐	☒	The statistical test(s) used AND whether they are one- or two-sided<br>*Only common tests should be described solely by name; describe more complex techniques in the Methods section.*
☐	☒	A description of all covariates tested
☒	☐	A description of any assumptions or corrections, such as tests of normality and adjustment for multiple comparisons
☐	☒	A full description of the statistical parameters including central tendency (e.g. means) or other basic estimates (e.g. regression coefficient) AND variation (e.g. standard deviation) or associated estimates of uncertainty (e.g. confidence intervals)
☐	☒	For null hypothesis testing, the test statistic (e.g. F, t, r) with confidence intervals, effect sizes, degrees of freedom and P value noted<br>*Give P values as exact values whenever suitable.*
☒	☐	For Bayesian analysis, information on the choice of priors and Markov chain Monte Carlo settings
☒	☐	For hierarchical and complex designs, identification of the appropriate level for tests and full reporting of outcomes
☐	☒	Estimates of effect sizes (e.g. Cohen's d, Pearson's r), indicating how they were calculated

Our web collection on statistics for biologists contains articles on many of the points above.

Software and code

Policy information about availability of computer code

Data collection	Data used to train the model were collected from the Uniprot database (Nov 17), searched using jackhmmer (hmmer 3.1b.1) (see methods) (all available on github), and nanobody sequences were collected from McCoy, et al, 2014. LINCLUST (MMseqs2 release 2-aecae) was used to cluster sequences for weighting.
Data analysis	SeqDesign is available on the github repository (https://github.com/debbiemarkslab/SeqDesign; DOI 10.5281/zenodo.4606785). Sequences were collected using jackhmmer. Codebase for training the model, predicting effects of mutations, generating sequences, and selecting a library is compatible with Python 2.7 or Python 3 and Tensorflow 1. For GPU-enabled computation, CUDA will have to be installed separately. For more details on analysis, see the methods section of this manuscript and the github repository. HMM comparisons were made using hmmer-3.2.1. FlowJo (10.6.1) and FlowCytometryTools v0.4.5 was used to analyze FACS data.

For manuscripts utilizing custom algorithms or software that are central to the research but not yet described in published literature, software must be made available to editors/reviewers. We strongly encourage code deposition in a community repository (e.g. GitHub). See the Nature Research guidelines for submitting code & software for further information.

Data

Policy information about availability of data

All manuscripts must include a data availability statement. This statement should provide the following information, where applicable:

- Accession codes, unique identifiers, or web links for publicly available datasets
- A list of figures that have associated raw data
- A description of any restrictions on data availability

Data collected for mutation scans from a wide range of primary sources (listed in Supplementary Tables 1, 2, and 4). All data generated and analyzed during the study are available in this published article, its supplementary information files and on the github repository (https://github.com/debbiemarkslab/SeqDesign). The data for pathogenic mutations in tau protein were collected from the Alzforum database: https://www.alzforum.org/mutations/mapt on Aug. 12, 2020. Comparisons for EVmutation, DeepSequence, and Indpendent models were made using reported data predictions in DeepSequence.

Field-specific reporting

Please select the one below that is the best fit for your research. If you are not sure, read the appropriate sections before making your selection.

☒ Life sciences ☐ Behavioural & social sciences ☐ Ecological, evolutionary & environmental sciences

For a reference copy of the document with all sections, see nature.com/documents/nr-reporting-summary-flat.pdf

Life sciences study design

All studies must disclose on these points even when the disclosure is negative.

Sample size	We used all available deep mutation scans from literature at time of analysis for blind, unsupervised mutation effect prediction.
Data exclusions	No data was excluded
Replication	The autoregressive model is run in replicates to verify predictions, with 3 replicates starting from different random seeds.
Randomization	Randomization is not relevant to the study for training the model because we do not need to split the data for training and test. We use random seeds for training, but these do not affect model performance.
Blinding	Blinding is not relevant to the study as the study is unsupervised learning, as we do not use any labeled data for training our models. Our method only uses natural sequences, and does not train on experimental data.

Reporting for specific materials, systems and methods

We require information from authors about some types of materials, experimental systems and methods used in many studies. Here, indicate whether each material, system or method listed is relevant to your study. If you are not sure if a list item applies to your research, read the appropriate section before selecting a response.

Materials & experimental systems

n/a	Involved in the study	
☐	☒	Antibodies
☐	☒	Eukaryotic cell lines
☒	☐	Palaeontology
☒	☐	Animals and other organisms
☒	☐	Human research participants
☒	☐	Clinical data

Methods

n/a	Involved in the study	
☒	☐	ChIP-seq
☐	☒	Flow cytometry
☒	☐	MRI-based neuroimaging

Antibodies

Antibodies used	anti-HA AlexaFluor647 conjugated antibody (Cell Signaling Technology) and synthetic nanobodies were used. Synthetic nanobodies (~200000 in the library) are cloned as designed from the computational model.
Validation	anti-HA antibodies were validated using antigen-non expressing cells to confirm minimal crossreactivity in experimental conditions. Synthetic nanobody characterization was performed using yeast display and induced, using FACS to measure expression and binding to HSA. A nanobody, Nb174684, was identified and sequenced. This process is described in more detail in the methods section of the manuscript,

Eukaryotic cell lines

Policy information about cell lines

Cell line source(s)	This information is included in the methods section of the manuscript.
Authentication	Yeast cell lines were validated by assessment of reported auxotrophic characteristics.
Mycoplasma contamination	Not relevant
Commonly misidentified lines (See ICLAC register)	Not relevant

Flow Cytometry

Plots

Confirm that:

☒ The axis labels state the marker and fluorochrome used (e.g. CD4-FITC).

☒ The axis scales are clearly visible. Include numbers along axes only for bottom left plot of group (a 'group' is an analysis of identical markers).

☒ All plots are contour plots with outliers or pseudocolor plots.

☒ A numerical value for number of cells or percentage (with statistics) is provided.

Methodology

Sample preparation	Yeast displaying the computationally designed or combinatorial synthetic nanobody library8 were grown in tryptophan dropout media with glucose as the sugar source for one day at 30 °C and then passaged into media with galactose as the sole sugar source to induce expression of nanobodies at 25 °C. After two days of induction, one million cells from each library were stained with a 1:25 dilution of anti-HA AlexaFluor647 conjugated antibody (Cell Signaling Technology) in Buffer A (20 mM HEPES pH 7.5, 150 mM NaCl, 0.1% BSA, 0.2% maltose) for 30 minutes at 4 °C. After staining, cells were centrifuged, the supernatant was removed, and cells were resuspended in Buffer A for flow analysis.
Instrument	Accuri C6 (BD Biosciences), SONY SH800Z Sorter for sorting
Software	BD Accuri C6 Software Version 1.0.264.21 was used to collect the data. FlowJo (10.6.1) and FlowCytometryTools were used to analyze the flow cytometry data.
Cell population abundance	Cell populations contained around 20-40% of cells that expressed nanobodies, as detected by the anti-HA antibody. When tested for binding to HSA (human serum albumin), proportions of cells that had detectable binding to HSA were identified.
Gating strategy	Standard gating was used. Yeast form a single population in an FSC/SSC plot and were gated accordingly. Cells were further gated on an FSC-A/ FSC-H plot to exclude doublets. A representative gating figure is included in Supplementary Figure 10.

☒ Tick this box to confirm that a figure exemplifying the gating strategy is provided in the Supplementary Information.
